# Primary Pericardial Sarcoma with Right Atrial Invasion and Multiple Bilateral Pulmonary Metastases in a Patient with Hereditary Nonpolyposis Colorectal Cancer

**DOI:** 10.1155/2016/6208029

**Published:** 2016-11-21

**Authors:** Eugene Wong, Lawrence J. Oh, Kazi Nahar, Adrian Lee, Stephen Clarke

**Affiliations:** Royal North Shore Hospital, Reserve Road, St Leonards, NSW 2065, Australia

## Abstract

Primary tumours originating from the pericardium are extremely rare. Previous studies have reported that these tumours account for only 6.7–12.8% of all mediastinal tumours with an overall prevalence of 0.001% to 0.007%. The majority of these tumours are benign lipomas or pericardial cysts. The most common pericardial malignancy is mesothelioma. Sarcomas are soft-tissue mesenchymal malignancies originating from various parts of the body but are extremely rare in this area. We report a case of a 52-year-old female who was diagnosed with a primary sarcoma with rhabdoid differentiation originating from the pericardium. The patient presented to her GP with a four-week history of progressive dyspnea and chest pain on exertion. Chest X-Ray demonstrated a prominent pericardial effusion and suspicious chest and pericardial lesions. Biopsies of the effusion and primary tumour identified on FDG/PET scans revealed the diagnosis of primary undifferentiated sarcoma. On thoracotomy, it was noted that the tumour had invaded the right atrium; therefore, pericardial window was aborted and a drain inserted instead. The patient was then started on chemotherapy; however, progression soon occurred and the patient died within 4 months, suggesting there is urgent need for efficacious treatments for sarcomatous lesions.

## 1. Background 

Sarcomas are rare malignant tumours that originate from connective or nonepithelial tissues. They affect many organs in the body, with the most common subtype being a Gastrointestinal Stromal Tumour (GIST) of the stomach. Sarcomas predominantly affect young patients, with a slight preponderance to males [1]. Sarcomas originating from the pericardium are even rarer entities, of which the most described histological subtype being an angiosarcoma, closely followed by undifferentiated sarcomas. A study in 2014 reported only 37 undifferentiated sarcomas that have been described in the literature with common presentations being dyspnea, fever, chest pain, and cough [2, 3].

## 2. Case Presentation

A 52-year-old female presented with a four-week history of progressive dyspnea on exertion and right-sided chest pain. The only other significant associated history was that she was diagnosed with localised Hereditary Nonpolyposis Colorectal Cancer in 2005 which was treated with colonic resection without adjuvant chemoradiotherapy. There was no asbestos or smoking history.

## 3. Investigations

**Figure 1:**
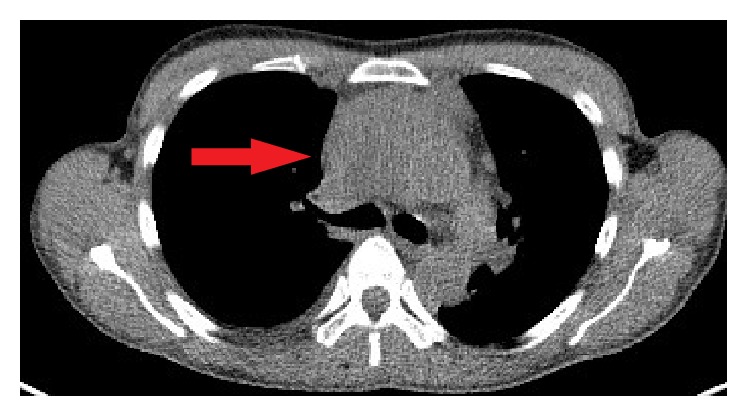
Axial CT demonstrating large anterior mediastinal sarcoma with involvement of the right atrium.

**Figure 2:**
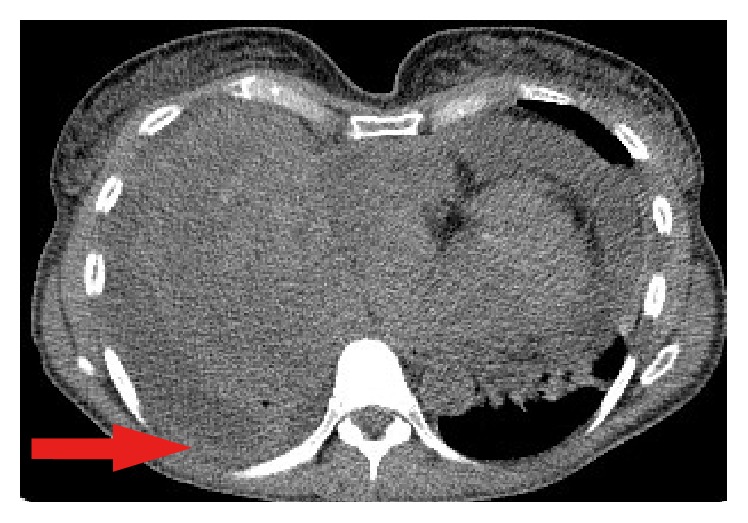
Axial CT demonstrating bilateral pulmonary effusions secondary to sarcoma.

A chest X-Ray was performed as the patient initially presented to her local GP, which demonstrated bilateral lung opacifications and a prominent pericardial effusion. This study prompted a high resolution computed tomography (CT) of her chest which demonstrated a large anterior mediastinal mass with extension of the right lower zone and abutting the right heart border. Images of the CT are demonstrated in Figures 1 and 2.

Mediastinal fine needle and core biopsies were then performed on percutaneous approach based on the above scans. Histopathology of a 20 × 25 × 6 mm lesion from biopsy revealed a malignant sarcomatoid tumour, with presence of PAX8, myogenin, myoD1, and desmin expression which suggests some skeletal or cardiac muscle differentiation. Flourescent in situ hybridisation studies failed to demonstrate gene rearrangements in SYT, EWSR1, DDIT (CHOP)/FUS, and FOX01/PAX3. INI1 was positive. Given these results, while the tumour represented some rhabdoid differentiation, it could not be further classified.

**Figure 3:**
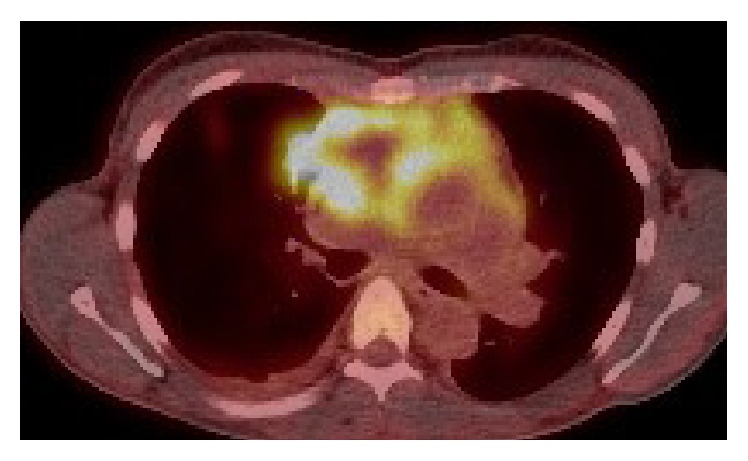
FDG PET demonstrating avid uptake at the pericardium and heart.

**Figure 4:**
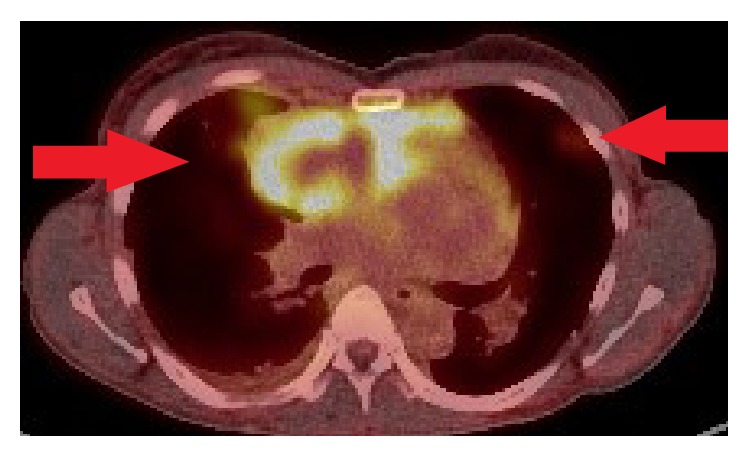
PET demonstrating FDG avid uptake in bilateral lungs, presumed metastases.

An FDG/PET scan demonstrated further focally increased uptake superiorly in the pericardial sac and multiple FDG avid pulmonary nodes scattered across all lobes of both lung fields (Figures 3 and 4).

Preoperative transthoracic echocardiography demonstrated a mild right atrial free wall collapse suggestive of early tamponade and a moderately sized pericardial effusion. A well circumscribed round mass was noted abutting the right atrial wall. There was otherwise normal biventricular size, wall thickness and function, and no significant valvular pathology.

## 4. Treatment

Given the above findings, this patient was at a high risk of tamponade from her pericardial effusion. Therefore, we decided to perform a thoracotomy to facilitate subxiphoid exploration of the pericardium. It was anticipated that a pericardial window procedure would be performed.

On thoracotomy, a solid tumour was identified involving the pericardium with invasion into the right atrium. Transoesophageal echocardiography performed at this time confirmed intra-atrial involvement of the tumour. The procedure was modified from a pericardial window to insertion of a pericardial drain because of these findings. The pericardial catheter was inserted percutaneously through a subxiphoid approach into the pericardial space lateral to the right ventricle.

During the above procedure, the patient developed atrial fibrillation (AF) with fast ventricular response, which was reverted to sinus rhythm after 20 mg of intravenous esmolol. The patient was extubated with no complications; however, on arrival to the Intensive Care Unit, she again developed rapid AF which reverted following administration of 300 mg Amiodarone. The patient thereafter had an uncomplicated recovery.

She then received postoperative first-line single agent Doxorubicin (75 mg/m2) with Pegfilgrastim commencing one week postoperatively, with a plan for continued 3-week treatment for a total of 6 cycles. The patient tolerated the first treatment well with no significant complications.

## 5. Outcome and Follow-Up 

The patient responded well following her second cycle of Doxorubicin chemotherapy and self-reported much improved breathing and quality of life compared to her postoperative level of function.

**Figure 5:**
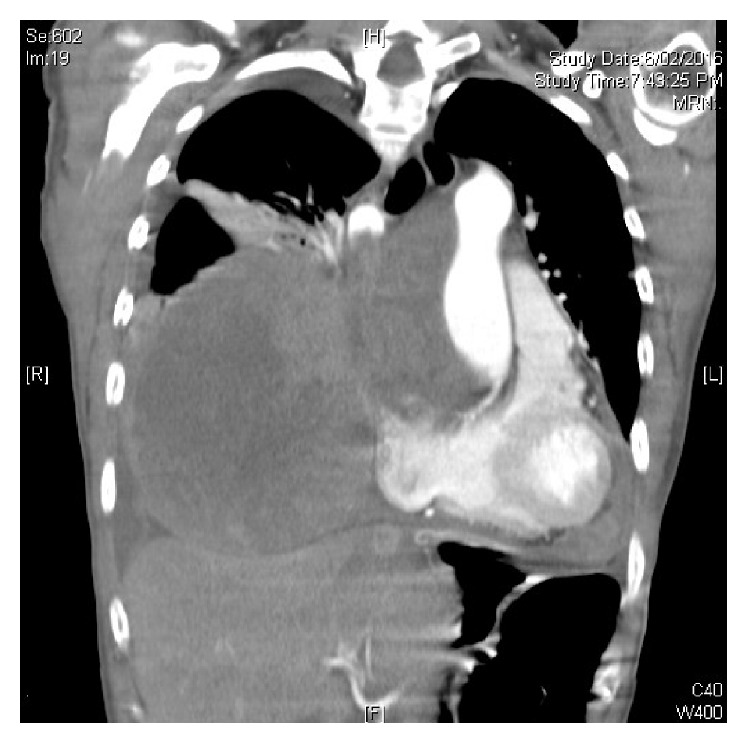
CT coronal 3 months following diagnosis demonstrating progression of disease at the Right atrium and Right lung.

**Figure 6:**
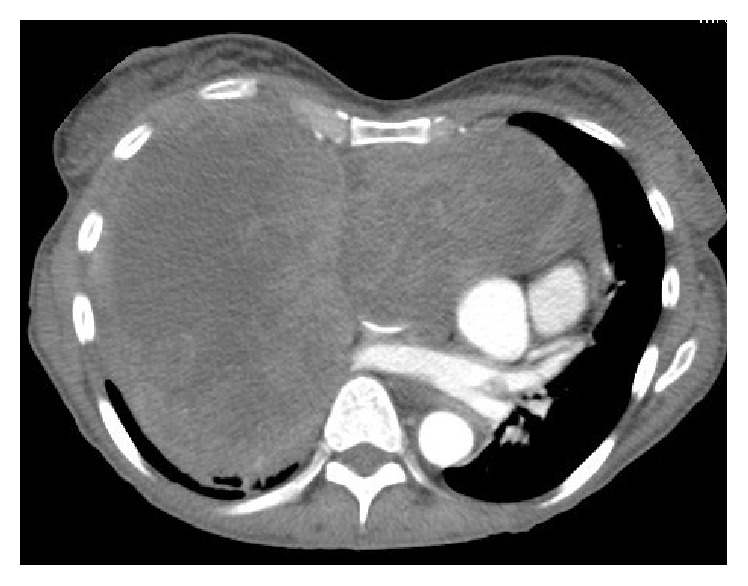
Axial CT at 3 months demonstrating progressive disease.

Unfortunately, three months later, the patient's disease progressed with significant worsening of the right pleural effusion and tumour pressure on the proximal left subclavian vein with development of a thrombus in the superior vena cava. There was also significant progression of the primary tumour. These findings were demonstrated on high resolution CT as shown in Figures 5 and 6. A decision was made at this stage to cease systemic therapy and for palliation of symptoms and the patient died two weeks later.

## 6. Discussion

Primary tumours originating from the pericardium or heart are extremely rare, with incidence at autopsy described at around 0.05% [4]. Those primary tumours that are malignant have reported autopsy incidence of around 0.001% [5]. The most common subgroup of malignant pericardial tumours are sarcomas, which are typically histologically mesotheliomas or angiosarcomas. Undifferentiated sarcomas are as such extremely rare, with previous reports suggesting that these may represent only 6-7% of sarcomas in this region. A systematic review published by Butany et al. demonstrated that approximately only 37 cases of undifferentiated sarcoma have been described from articles dating back to 1957 [3].

In our case, the patient presented with a suspected pericardial tumour following investigations of dyspnea secondary to malignant pericardial effusion. The patient underwent both a diagnostic and therapeutic insertion of pericardial drain and postoperatively experienced a recognised complication of cardiac arrhythmia but did not experience other potential complications such as ventricular puncture or pneumothoraxes [6].

This case is interesting for a number of reasons. Firstly, the pathological entity is rare, particularly due to its right-sided pericardial origin and extension into the right atrium, which required modification of surgical intervention from a pericardial window to drain insertion only. Furthermore, this is also the first case to our knowledge of an association between sarcoma of this region and a history of Hereditary Nonpolyposis Colorectal Cancer, although its relevance is uncertain.

The prognosis for metastatic sarcomas of the pericardium and heart is poor, often because of incomplete surgical resection and that radiotherapy and chemotherapy only offer temporary improvement. Gibbs et al. reported that median survival for these patients is around 5 months [6].

Extending the discussion to all localised soft-tissue sarcomas that present in adulthood, there is conflicting evidence surrounding the efficacy of chemotherapy in the adjuvant setting and remains a controversial topic. As such, wide-local surgical excision with adjuvant radiotherapy remains the cornerstone of treatment in these patients [7]. Unfortunately, our patient had myocardial tumour invasion and distant metastases and therefore neither of these treatment options were amenable.

As this patient was initially experiencing significant shortness of breath for four weeks that limited her function substantially, we felt that she experienced a good response to the early initiation of Doxorubicin chemotherapy. Although there was some symptomatic benefit from initial chemotherapy, progression soon occurred and the patient died within 4 months, suggesting there is urgent need for new treatments for sarcomatous lesions.
